# Effects of central-peripheral FMS on urinary retention after spinal cord injury: a pilot randomized controlled trial protocol

**DOI:** 10.3389/fneur.2023.1274203

**Published:** 2024-01-05

**Authors:** Lingyan Dong, Xi Tao, Cheng Gong, Yi Long, Li Xiao, Yun Luo, Maoyuan Wang, Yanbiao Zhong

**Affiliations:** ^1^Gannan Medical University, Ganzhou, Jiangxi, China; ^2^Department of Rehabilitation Medicine, The First Affiliated Hospital of Gannan Medical University, Ganzhou, China; ^3^Ganzhou Key Laboratory of Rehabilitation Medicine, Ganzhou, China; ^4^Ganzhou Intelligent Rehabilitation Technology Innovation Center, Ganzhou, China; ^5^Key Laboratory of Prevention and Treatment of Cardiovascular and Cerebrovascular Diseases, Ministry of Education, Gannan Medical University, Ganzhou, China

**Keywords:** spinal cord injury, urinary retention, urination diary, residual urine volume of the bladder, repeated magnetic stimulation, fNIRS

## Abstract

**Background:**

Urinary retention is a common complication of spinal cord injury (SCI), which can seriously affect the quality of life of patients. Function magnetic stimulation (FMS) has been widely used in the recovery of neurological function in various diseases, but its application in urinary retention after SCI remains unclear. Therefore, we would like to conduct a pilot randomized controlled trial (RCT) to observe the feasible effect of FMS on urinary retention after SCI, to explore its mechanism of action.

**Method/design:**

This is a single-center pilot RCT, which 60 patients with urinary retention after SCI will be selected, numbered in chronological order of hospitalization, and randomly divided into 4 groups using the random number table method, Groups A (control group), Group B, Group C, and Group D; Each group will receive the same conventional rehabilitation treatment. The whole intervention period 2 weeks and will be evaluated before and after treatment to collect data on residual bladder volume, functional near-infrared spectroscopy (fNIRS), changes in voiding condition, changes in surface electromyography (SEMG) values of pelvic floor muscle and quality of life scores (QoL).

**Study hypothesis:**

We hypothesized that FMS for the treatment of urinary retention after SCI would have a significant clinical feasible effect;and that peripheral combined with central FMS would be more effective than single-site FMS for the treatment of urinary retention after SCI.

**Objective:**

(1) To illustrate the clinical effectiveness of FMS in the treatment of urinary retention after SCI and to provide a new treatment modality for the patients; (2) Comparison of the differences in the efficacy of central and peripheral single FMS and combined central and peripheral FMS in the treatment of urinary retention after SCI; (3) To explore the central control mechanisms of bladder function recovery after SCI in conjunction with changes in fNIRS.

**Trial registration:**

This study has been ethically approved by the Scientific and Ethics Committee of the First Affiliated Hospital of Gannan Medical university with approval number (LLSC-2022112401). It has been registered with the China Clinical Trials Registry with the registration number: ChiCTR2200067143.

## Introduction

### Background and rationale

Spinal Cord Injury (SCI) is a serious and disabling disease. The global prevalence of SCI is 27.04 million, with an incidence rate of 130 per 100,000, and the incidence of SCI is increasing (1, 2). More than 80% of patients with SCI have varying degrees of bladder dysfunction (3). Urinary retention is a common complication after SCI and is characterized, by bladder distension, inability to urinate or frequent urination, feeling of incomplete urination, lower abdominal distension and discomfort. It is easy to cause urinary tract infection, hydronephrosis and even life-threatening renal failure, which brings great inconvenience to the daily life of patients with SCI and seriously reduces the quality of life (4, 5).

At present, domestic and international treatments for urinary retention after SCI mainly include catheterization, medication, surgery, traditional rehabilitation training, electrical stimulation and so on (6). Among them, long-term indwelling catheterization is prone to urinary tract infection, urethral injury, and bladder atrophy (7); Long-term drug treatment leads to drug resistance in some patients and more adverse drug reactions; surgical treatment may lead to disorders of bowel fluid secretion, urinary incontinence, postoperative incision infection (8, 9); Traditional rehabilitation training has inconsistent results and variable treatment effects (10); Electrical stimulation carries the risk of discomfort and accidental injury (11). These treatments are not ideal, therefore, there is an urgent need to find a new treatment to further improve the clinical efficacy of patients with urinary retention after SCI.

Function magnetic stimulation (FMS) is a kind of treatment that generates a magnetic field through a coil, which further generates an induced electric field to depolarize the nerve tissue, so as to stimulate the nerve or muscle and produce an excitatory or inhibitory neuromuscular effect. It has the advantages of being painless, noninvasive, simple and easy to use, and is currently used clinically to treat psychological disorders, stroke, and SCI at present (12, 13). With the development of FMS therapy in recent years, many researchers have applied FMS therapy to urinary retention after SCI and reported positive clinical outcomes (14). According to the functional application of FMS, it is currently divided into peripheral FMS and central FMS, and the commonly used stimulation mode is repetitive function magnetic stimulation (rFMS). When rFMS is applied to a peripheral nerve or muscle, it is called repetitive peripheral magnetic stimulation (rPMS). When rFMS is applied to the cortex, it is known as repetitive transcranial magnetic stimulation (rTMS). Common used peripheral sites include sacral nerve roots, pelvic floor perineum, suprapubic bladder area, etc. Among them, stimulation of S3 nerve roots is more common (15, 16). Sacral nerve root MS can induce contraction of both the urethral and pelvic floor muscles, thus facilitating the production of urine flow; when the stimulation stops, the transverse urethral sphincter will relax immediately, while the urethral muscle is still markedly tense, and the pressure difference between the bladder and the urethra allows the urine to be discharged (17). Sacral nerve root MS excites contraction of the urethral muscle due to delayed activation of the spinal micturition reflex pathway, which excites the parasympathetic nerves and induces micturition (17). MS of the sacral nerve roots also activates peripheral afferent nerve fibers to enhance sensory input to the spinal cord, increasing the excitability of the descending corticospinal tracts and further inducing contraction of the urethral muscles (18). Rodic et al. (19) showed that high frequency rMS of S3 nerve roots could promote the contraction of bladder force muscle in patients with urinary retention, increase bladder pressure, and promote urination. At present, the central stimulation target is the primary motor cortex (M1), High-frequency FMS stimulation of the M1 region can activate the pontine micturition center (PMC), the neural nucleus associated with micturition found in the subcortical structures of the cerebral cortex, to enhance the activity of the urethral muscles, promote bladder contraction, and induce voluntary voiding (20). At the same time, rFMS can induce and enhance the neural conduction of other subcortical regions through stimulation of the M1 region, so that the patient can perceive the filling of the bladder, and thus can independently control the voiding reflex to maintain an effective storage and voiding state (21). Cortical high-frequency rFMS can also effectively promote the pubic-anal reflex in patients with incomplete spinal cord injury, improve the function of pelvic floor sphincter (22), strengthen the contraction of pelvic floor muscles, and improve the ability of urinary control.And the L5 layer neurons in M1 region are closely related to urination behavior. Activation of L5 neurons in M1 region will trigger bladder contraction and urination. Therefore, MS in the M1 region of the cortex can not only activate L5 neurons in the M1 region, but also use its remote effects to regulate PMC, thereby improving urination function and improving the quality of life of patients (23–25).

In the past few decades, with the development of imaging techniques, it has been possible to obtain imaging data on brain function by measuring metabolic function and blood flow changes in cortical areas that indirectly reflect local neural activity. Studies showed that vertebral cells in the primary motor cortex (M1) undergo different levels of trophic death determined by the segment and type of injury, which altered the response of the motor cortex to stimuli above the focal segment, resulting in changes in the cortical network, leading to a series of structural and functional alterations of the brain tissues controlling and transmitting neural signals, (26, 27) that is, changes in brain plasticity. Functional magnetic resonance imaging (MRI), with its advantages of non-invasive and repeatability, can continuously observe the plasticity of brain function in patients with SCI and provide imaging basis for clinical treatment. Previous studies on brain functional imaging techniques of SCI and urination dysfunction have shown (28, 29) increased neuronal activation in the bilateral primary motor cortex (M1), primary sensory cortex (S1), auxiliary motor area (SMA), paracentral lobule and other areas after SCI. The higher center of urination reflex involves many brain regions, including pons, periaqueductal gray matter, thalamus, prefrontal cortex, cingulate cortex, auxiliary motor area. Due to the limitations of research methods and conditions, the imaging studies of brain-bladder regulation are still in the initial stage.Near infrared functional brain imaging, also known as functional near-infrared spectroscopy (fNIRS), is a new type of functional brain imaging technology based on the neurovascular coupling mechanism, using the optical principle to detect the spectral changes caused by hemoglobin. fNIRS has the advantages of low cost, portability, high interference resistance, and high temporal resolution (30). fNIRS has been gradually, applied to hypothesize the mechanism of bladder control center (28), Because of its ability to easily detect changes in brain function after treatment, it can further guide neuromodulation therapy of neurogenic bladder according to the relevant indicators such as electrophysiology of the bladder, bladder capacity.

There is a lack of studies comparing the advantages and disadvantages of the effects of central and peripheral FMS for the treatment of urinary retention after SCI, and the effects of central combined with peripheral FMS for the treatment of urinary retention after SCI have rarely been studied. Therefore, the aim of this study is to compare the feasible effects of central and peripheral FMS in the treatment of urinary retention after SCI and to explore its mechanism of action and to provide reference for the clinical treatment of patients with urinary retention after SCI.

## Methods and study design

### Study design

**Figure 1:**
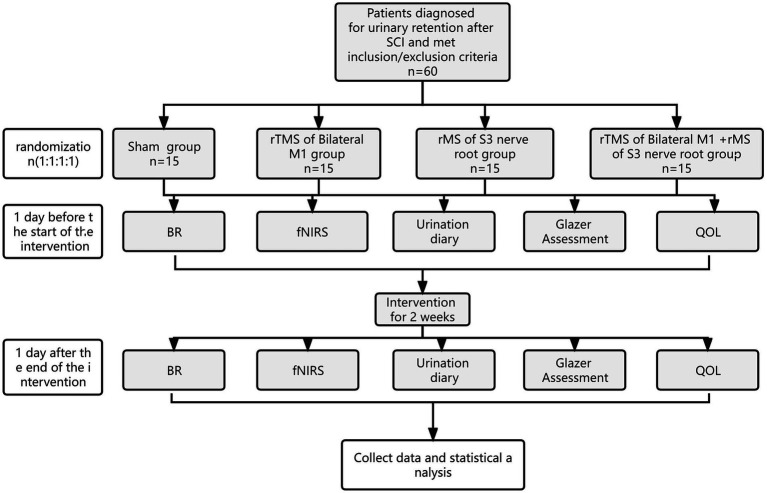
Study design. SCI, spinal cord injury; rMS, repeated magnetic stimulation; BR, bladder residual urine; fNIRS, functional near-infrared spectroscopy; QoL, quality of life.

This trial is a pilot single-center, prospective, double-blind, randomized, controlled study (e.g., Figure 1), and 60 patients with urinary retention after SCI will be selected. According to the sequence of hospitalization time, patients will be randomly assigned to group A (sham stimulation), group B (rTMS of Bilateral M1), group C (rMS of S3 nerve root), and group D (rTMS of Bilateral M1 + rMS of S3 nerve root) in a 1:1:1:1 ratio, combined with conventional rehabilitation treatment.The intervention measureswill be once a day, 20 min, 5 times a week, and the whole experiment cycle will last 2 weeks (10 times in total). Study time point: bladder residual urine volume, brain fNIRS, urination diary, pelvic floor surface electromyography Glazer assessment and quality of life (QoL) assessment will be performed 1 day before treatment and 2 weeks after treatment, respectively. A flowchart overview of the study is presented in Figure 1.

This study will obey the Standard Protocol Items: recommendations for Interventional Trial (SPIRIT) guidelines (31). Each participant in this study will sign an informed consent form (Supplementary material) prior to participation. All participants will be informed of the nature of the trial, the purpose and procedure involved, the expected completion time, potential adverse reactions and possible benefits.

### Ethics and dissemination

This study was approved by the Science and Ethics Committee of the First Affiliated Hospital of Gannan Medical University on November 30, 2022 (Approval number LLSC-2022112401), and has been registered in the Chinese Clinical Trial Registry (registration no. ChiCTR2200067143, https://www.chictr.org.cn/index.html).

## Eligibility criteria

### Inclusion criteria

Participants who met all of the following criteria willbe considered for inclusion: (1) met the classification criteria for SCI in the International Standard for Neurological Classification of SCI (32) and were confirmed by MRI or CT; (2) clinically present with varying degrees of urinary retention, i.e., residual urine volume > 100 mL after bladder emptying; (3) aged 18–60 years, regardless of gender; (4) patients graded B, C, or D according to the American Spinal Cord Injury Association functional rating criteria (ASIA); (5) had a disease duration between 1 month and 2 years and underwent intermittent catheterization; and (6) voluntarily participatingin this study and signingan informed consent form.

### Exclusion criteria

Patients with any of the following conditions will be excluded: (1) concurrent urinary tract infection, malignant tumor, stone.; (2) Patients with previous history of epileptic seizure, central nervous system disease, mental disease, and psychological disease; (3) Patients with cranial and sacrococcygeal metal implants, increased intracranial pressure, heart disease, cardiac pacemaker, drug pump, and unable to receive MS treatment; (4) Patients who are taking tricyclic antidepressants, neuroleptics, or have a family history of epilepsy; (5) Dysfunction of urination caused by other diseases; (6) Patients who had undergone urethral sphincterotomy or cystostomy; (7) Patients with unstable vital signs, hemodynamic instability, skin damage, and bleeding tendency; (8) Pregnant and lactating women.

### Drop out criteria

Once subjects have signed the informed consent form and have been screened and are eligible for entry into the clinical trial, cases that do not complete all clinical trial visits, regardless of the reason, will be considered to be disengaged from the clinical trial as long as they do not complete the clinical trial. If the patient develops urinary tract infection and is given the appropriate treatment, and the infection can be controlled within 1 week, the patient will be allowed tocontinue to complete the experiment; Patients who terminate the trial because their condition has resolved and no further treatment is required will not be included in the shedding.

### Sample size calculation

According to published literature (33) the mean post-intervention residual urine volume in the four groups A, B, C, and D were expected to be 302.6, 56, 107.2, and 179, respectively, with a standard deviation of 160.4. The degree of certainty 1−β = 0.90, test level *α* = 0.05, group A:B:C:D = 1:1:1:1 and sample size estimation will be performed using PASS 15 software according to the following equation.


$$n=\Psi^{2}\left[\overset{k}{\underset{i=1}{\sum }}{s_{i}}^{2}/k\right]/\left[\overset{k}{\underset{i=1}{\sum }}{\left({\bar{X}}_{i}-\bar{X}\right)}^{2}/\left(k-1\right)\right]$$

According to the predefined parameters, the minimum required samples for groups A, B, C and D were all 12 cases after the calculation of residual urine volume using One-Way Analysis of Variance F-Tests under the Means menu of PASS 15 software. Considering the lost rate of 20%, it is expected that 15 cases will be included in groups A, B, C, and D in this study, and a total of 60 cases will be included, which can ensure the accuracy and scientific accuracy of the study results.

### Recruitment strategies

Eligibility screening of SCI patients will receive rehabilitation treatment at the Department of Rehabilitation Medicine, The First Affiliated Hospital of Ganan Medical University, from January 2023 to December 2023 will be conducted in strict accordance with the inclusion and exclusion criteria mentioned above, those patients who pass the eligibility screening will be included in the randomized trial. If a participant withdraws midway through the trial, the reason for withdrawal will be carefully documented. During the recruitment process, potential participants will be briefed on the intervention approach, study schedule and study procedures. The Standard Protocol Items: SPIRIT table for enrolment, interventions, and assessments is presented in Table 1.

**Table 1:** Schedule of enrollment, assessments, and interventionis in the RCT.

	Screening	Baseline evaluation	0	2 week	Post evaluation
**Enrollment**					
Informed consent	×				
Demographic information	×				
Vital sign	×				
Medical history	×				
Eligibility assessment	×				
Random allocation		×			
**Allocation Assessments**					
fNIRS		×			×
Residual bladder urine volume		×			×
Urination diary		×			×
Glazer assessment		×			×
QOL		×			×
Interventions					
Sham group			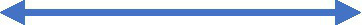		
M1 RMS group			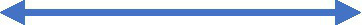		
S3 RMS group			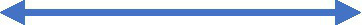		
M1 + S3 group			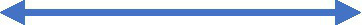		

“×” indicates at which point of the RCT the respective assessments will take place 0: time of the start of treatment baseline evaluation: one day before treatment post evaluation: 2 weeks after treatment and evaluation.

### Randomization and blinding

Randomization of included patients based on length of stay will be performed using the random number table method. Throughout the study, all investigators and subjects will be unaware of the allocation of interventions, Outcome evaluators and data analysts will be blinded, and subjects will be randomly assigned to groups by persons unrelated to the trial. Opaque closed envelopes containing sequence numbers used for allocation concealment will be identified with different numbers. Once the sequences is generated, the envelopes will be placed in a secure place in numerical order and the paper containing the corresponding group information will be placed in the envelopes. Sequence generation and production will be performed by rehabilitation physicians not involved in this study.

An experienced blinding rehabilitation physician or therapist will perform an initial assessment of enrolled patients and an assessment after 2 weeks of treatment. In the event of a serious adverse event, emergency unblinding will be performed and the involved subject will be removed from the trial protocol. When the experiment is complete, the statisticians will have four sets of data.

### Research group and intervention

1. In this experiment, Wuhan Ired MagTD 60 magnetic stimulator will Be used, The stimulation coil will select a circular coil, and The stimulation parameters will Be selected As follows: the stimulation frequency will choose 20HZ, The intensity will select 80–120% resting motion threshold (RMT), The stimulation time will last for1s, The interval will last for9s, and a total of 20 Min will Be stimulated once a day, 5 times a week for 2 weeks.

Group A: in the sham stimulation group, subjects will remain in a supine position, and the stimulation coil will be chosen to be a circular coil with the stimulation site 2 cm in front of the vertex, which is not discharged, and another “8” coil rotated and placed under the subject’s pillow to avoid scalp stimulation (34).

Group B: rTMS of Bilateral M1, The patients will beseated or supine, wore anatomic positioning caps, and selected M1 region according to the international EEG 10–20 system (25), and the handle willpointed to the anteromedial aspect of the sensorimotor cortex on one side, the neck will beimmobilised to avoid head movement and the coil was placed close to the scalp with the central stimulation point tangential to the scalp. To ensure the accuracy of the MS site, the patient’s RMT will be determined by a single manual stimulation, and alternating left and right stimulation was given for 10 min each according to the stimulation parameters.

Group C: rMS of S3 nerve root, the patient will beplaced in prone position, and the stimulation site will select the S3 nerve root region (about the midpoint of the upper sacral edge and the tailbone line to the left and right). The center of the coil will be as close to the surface of the sacrum as is appropriate. Stimulation will be determined by bilateral toe movements or pronounced anal contractions (35), identified as RMT, and then rMS will be given based on the stimulation parameters.

Group D: dual-target stimulation of bilateral M1 and S3 sacral nerve roots will be performed at the same time.

2. All four groups of patients will be given bladder care and conventional rehabilitation treatment. Bladder care: ① Drinking plan: patients will berequired to have a daily fluid intake of 1,550–1,650 mL, not exceeding 2,000 m l, and evenly distributed between 8:00–20:00 (drinking once every 2 h), including the amount of fluid flow and the amount of intravenous fluid input. ② Intermittent clean catheterization (36): each catheterization will be preceded by self-catheterization, and if it is not possible or there is residual, then catheterization will be performed. The frequency of intermittent catheterization is decided according to bladder capacity and residual urine volume, up to 6 times per day. Conventional rehabilitation treatment: ①Bladder function training (37): finger tapping on the suprapubic area to find the trigger point, and for patients who cannot find the trigger point, uniform Percussion on the bladder area 2–3 cm above the pubic symphysis, 50–100 times/min, 2–3 min percussion each time. ②Acupuncture treatment Acupuncture treatment will be performed by an experienced Chinese medicine practitioner who has been practicing acupuncture for more than 5 years, selecting the points Shangxing, Baihui, Qihai, Guanyuan, Zhongji, Baxiao, Double Large Intestine Yu, Double Kidney Yu, Double Guanyuan Yu, Double Bladder Yu, Double Chichibian, Double Huanjiu, Double Chengfu, Double Huizhong, Double Ashigangsanli, Double Sanyinjiao, Double Yinlingquan, Double Fenglong, Double Hangzhong, Double Kunlun, and clean and disinfect each point; and then needle the acupuncture points and leave the needles for 30 min. The treatment will be performed once a day for 30 min, 5 times a week for 2 weeks.

## Outcome measures

Outcome measures will be evaluated at 1 day before intervention and 2 weeks after the start of treatment. All evaluations will be conducted by the same uninformed therapist and rehabilitation physician.

### Primary outcome

The most important evaluation index is the residual urine volume of the bladder, the residual urine volume of the bladder can intuitively show the improvement of the urination function of patients with urinary retention. The main way is to measure the residual urine volume of the urinary system color ultrasound of the four groups of patients on the first day of treatment and the first day after treatment. Prior to the examination, the patient first attempts to empty the urine without external force or manipulation, and then a urinary ultrasound will be performed immediately. During the examination, the patient will be asked to lie flat on their back with the probe located in the midline above the pubic symphysis. Each measurement will scan the bladder in both the sagittal and transverse planes and measure the bladder capacity at this point to get an idea of the patient’s residual urine volume. The smaller the residual urine volume, the lower the degree of urinary retention.

### Secondary outcomes

#### Urination diary

The urination diaries will bea secondary outcome of this study. The urination diaries of 3 days before and after treatment will be recorded in this study. When recording the urination diary, it will berequired to ensure that the recording duration of a day is continuous for is 24 h, and to ensure the accuracy of the diary. The fluid intake will berecorded in units accurate to milliliters, and the volume of urine would measurusing a professional urine measuring tool to demonstrate the accuracy of the urination diary. The overall improvement of urinary catheterization times, voiding times, voiding volume and residual urine volume in the four groups for 3 consecutive days before and after treatment will beevaluated, and the mean value was calculated. In patients with urinary retention, the number of catheterizations decreased, the number of urination increased, and the volume of urination and residual urine increased, indicating that the symptoms of patients improved more significantly.

#### Functional brain imaging techniques

**Figure 2:**
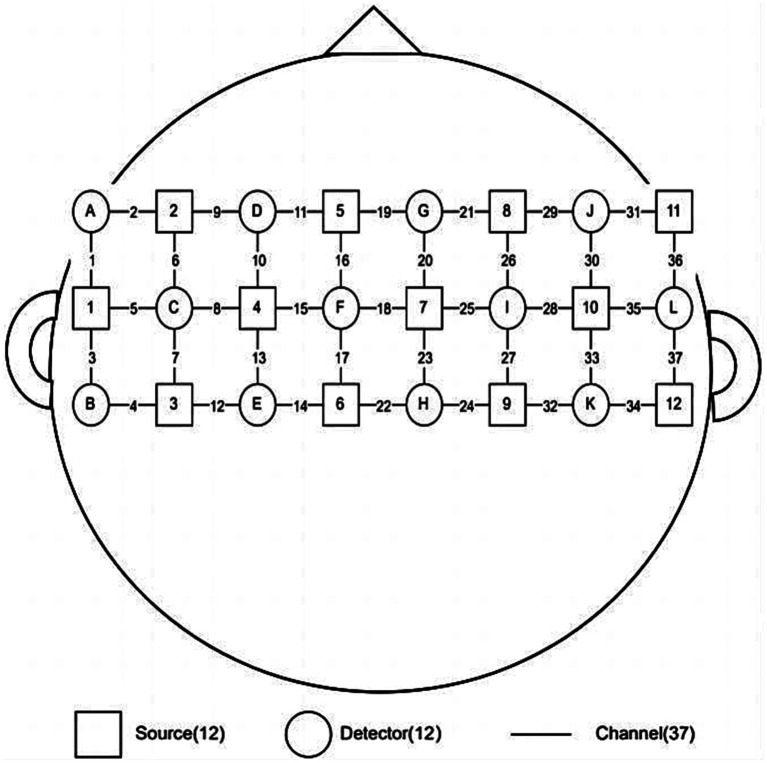
Channel layout diagram.

In this study, we will collecte the fNIRS data with a 37-channel device (BS-3000, Wuhan Zilian Hongkang Technology, Wuhan, China), and will use BrainScope software to display and save the fNIRS data. The data will be processed by Wuhan Yiride Medical Equipment Co., LTD, and the average concentration changes of oxy-hemoglobin (HbO2), Deoxy-hemoglobin (HbR) and Total- hemoglobin (HbT) in each channel of subjects in each group will be calculated. Before the experiment, the patients will be asked to sit in a chair, and the light source and detector will be placed on the head and close to the scalp of the patients. The signals will be mainly collected in the primary motor area (M1), pre-motor area and auxiliary motor area, in which channel 19 will beused as the Cz reference point, and the left and right cerebral hemispheres were distributed symmetrically, As shown in Figure 2. The main detection indexes were the content of HbO2, HbR, and HbT. Participants will berequired to keep quiet during the test without any movements (29). The block design was adopted: the resting period was 25 s and the task period was 15 s. Considering the need for subjects to acclimatize to the detection environment and to ensure that the fNIRS signal reaches a steady state, 25 s of baseline resting data will be collected before proceeding to the task period. The resting period and the task period will be repeat for 5 times, and then the resting data of 25 s will be collected after the end, for a total of 225 s. Resting period (25 s): 25 resting periods in which participants rest without any movement. Task period (15 s): complete the “anal lift” 3 times within 15S according to the operator’s suggestion (the “anal lift” movement is guided by the pelvic floor rehabilitation therapist before the test).

### Glazer pelvic floor surface electromyography (sEMG) assessment

Glazer pelvic floor sEMG assessment is the gold standard for evaluating pelvic floor muscle function, which can evaluate pelvic floor muscle support function, sexual function and sphincter function to understand the effect of pelvic floor muscle rehabilitation. The pelvic floor EMG values will be measured on the first and last day of treatment: before the assessment, the patient will be placed in a comfortable and relaxed position, in a truncated position, with the upper and lower body at a certain angle (about 120°), and the feet were naturally externally rotated to avoid the interference of the contraction of the intrapelvic muscles with the pelvic floor EMG signal. The electrodes will beplaced in the vagina (or anus) with metal pieces touching the pelvic floor muscles on the left and right sides to collect pelvic floor surface myoelectricity; the abdomen will beplaced under the umbilicus with disposable body electrode pieces to detect the force of abdominal muscles and avoid abdominal muscle compensation.

#### Quality of life (QoL)

By understanding the subjective perception of the impact of bladder symptoms on the quality of life (38), it can reflect the severity and efficacy of neurogenic bladder symptoms. By asking the patient, “What do you think if you continue to have the same urination symptoms for the rest of your life?” According to the patient’s satisfaction with the current urination symptoms, the answer was: 0: happy; 1: Satisfaction; 2: Generally satisfied; 3: It’s OK; 4: not satisfied; 5: Distress; 6: It’s bad. The higher the score, the worse the patient’s quality of life.

### Statistical analysis

All data will beanalyzed using SPSS 21.0 statistical software. The measures will beexpressed as mean ± standard deviation ( $\bar{x}$ ±SD), and one-way analysis of variance (ANOVA) or chi-square test will be used in all demographic characteristics and baseline assessments, The primary and secondary outcome measures will be expressed as $\bar{x}$ ±SD, and the data will be tested for a normal distribution. Before and after treatment, the uniformity of variance data between the two groups will be analyzed using *t* test. If the data is skewed, it will be converted to normal distribution according to the data distribution type; If the data does not conform to any distribution law, the rank sum test will be used to test the hypothesis of the data. Data with abnormal distributions or uneven variances will be analyzed using Wilcoxon rank sum tests. *X*^**2**^ test was used for counting data, and *p* < 0.05 for all tests was considered statistically significant.

The fNIRS data will beprocessed by professional data analysts of Wuhan Ired Medical Equipment Co., LTD. SPSS 21.0 will beused for data analysis to calculate the differences in activation intensity of each brain region, and the average concentration changes of HbO2, HbR, and HbT in each channel of subjects in each group will becalculated. Paired sample *t* test will beused to investigate the differences in blood oxygen changes before and after treatment, and one-way analysis of variance will be used for comparison between groups. *p* ≤ 0.05 will beconsidered as a statistically significant difference.

### Safety and adverse effects of rFMS

RFMS is generally noninvasive and safe, but occasionally causes transient headache and fatigue (39). Adverse events will be managed as follows: (1) If informed of discomfort, the investigator will immediately stop treatment, assess the patient’s vital signs, type and degree of discomfort, and then administer appropriate treatment. (2) All adverse events will be recorded and reported to the principal investigator and the ethics committee, who will jointly decide whether the patient is fit to continue participation.

### Post-trial care

If an adverse event occurs to a patient during the trial, the investigator in charge of the study will provide timely treatment to the patient. For injuries caused by this study, the principal investigator and the First Affiliated Hospital of Gannan Medical University will pay the relevant medical expenses and provide appropriate financial compensation in accordance with relevant national laws and regulations. Subjects included in the control group will receive conventional rehabilitation treatment to ensure effective spinal cord injury rehabilitation during the study period.

### Data integrity and management

All study data and records will be stored and managed in an electronic database. Paper files will be kept under seal. Only the researchers and statistical experts involved in the study will have access to the database.

### Original data collection plan and data entry

Raw data will be collected directly using paper forms or assessment equipment. The data for each form will be entered into a computer by two independent people. The data collected by the evaluation equipment will be classified and entered by two independent people. The data entered by the two people will be compared using SPASS 26 software. Any inconsistencies will be investigated and corrected.

### Auditing

After the audit is complete, the database will be backed up. Principal investigators, statisticians and supervisors will lock down the data to ensure data security. Any changes to the database after the audit can only be made with the consent of all parties.

### Withdrawals

Participants may withdraw from the study due to serious adverse events (such as seizures or cerebrovascular accidents) or other personal factors (participant refusal to continue treatment, etc.).

### Ethics and dissemination

Any protocols or documents that need to be modified during the trial will be reviewed by the Ethics Committee. In addition, the study team will report the progress of the project and other relevant matters to the Ethics Committee periodically during the trial period, and the Ethics Committee will review all trial data.

### Consent

The study team member responsible for recruiting patients will explain in detail to the patient the main elements of the study and all items of the informed consent form to the patients in detail. Any questions asked by potential recruits will be answered. Subjects who agree to participate in the study will sign the informed consent form to indicate their participation. Since only Chinese participants will be recruited, all study documents will be in Chinese. No biological samples will be collected from participants and therefore no signed documents are required.

### Confidentiality and data access permissions

We will protect the privacy of patients to the extent permitted by law. Any public reporting of the results of this study will not disclose any personal patient information. Identifying information will not be disclosed to individuals outside the study team without the patient’s permission. All study members will be required to maintain the confidentiality of the patient’s identity and associated personal data.

## Discussion

After SCI, the loss of contact between the higher voiding centers in the brain, that control the bladder and the lower voiding centers in the spinal cord cause voiding or urinary storage disorders, manifest by urinary retention, urinary incontinence, or a mixture of both, which severely affects the quality of life of patients (40). The most common cause of death in lower urinary tract voiding disorders after spinal cord injury is renal failure due to long-term recurrent vesicoureteral reflux caused by urinary retention (41, 42). Therefore, improving urinary retention symptoms and reducing upper urinary tract reflux are the most important measures to protect renal function. Currently, the rehabilitation for the treatment of urinary retention after SCI is unsatisfactory, and there is an urgent need for new treatments with definitive efficacy.

MS is the current research hotspot of non-invasive neuromodulation rehabilitation technology at home and abroad. The principle of its use is to use a time-varying magnetic field to generate an induced electric field, which passes through the bone, fat and skin without attenuation, so that the action site generates an induced current and finally achieves the effect of excitation or inhibition of neuromuscular. RMS is clinically used for the treatment of stroke, cerebral hemorrhage, SCI, peripheral nerve injury, and psychiatric disorders because of its non-invasive, painless, and easy features (1). The core area of the motor cortex, M1 area can enhance the nerve conduction in other subcortical areas, such as the periaqueductal gray matter area, thalamus and insula, etc., which can control the corresponding muscles of the spinal cord and regulate the neural pathways, thereby addressing voiding dysfunction (25, 43).

Studies have shown that high-frequency rMS of area M1 can promote bladder contraction, reduce maximum bladder capacity, increase voiding volume, achieve low-pressure urinary storage, and create periodic voiding (44). A study by Centonze (45) used rTMS at a frequency of 5 Hz to treat patients with multiple sclerosis voiding dysfunction and found that enhancing corticospinal tract excitability may help improve bladder dysfunction. MS of the sacral nerve has a bidirectional modulatory effect that coordinates the relationship between the bladder forcing muscles and urethral sphincter, improving the voiding and urinary storage function of patients (12). Among them, S3 nerve root stores the parasympathetic nerves of the detrusor muscle and sensory and motor fibers of the pelvic floor muscle. a MS can directly excite parasympathetic nerve or stimulate sensory and motor fiber conduction of the pelvic floor muscle to promote voiding (46–48). Rodic et al. (19) used 20 Hz FMS stimulation of S3 nerve root to treat posterior detrusor pressure in patients with urinary retention after SCI.

The above studies have shown that MS of the central M1 area and S3 nerve root can improve the symptoms of patients with urinary retention after SCI, but there are few studies comparing the advantages and disadvantages of MS of the M1 area or S3 nerve root in treating patients with urinary retention after SCI and the effects of combined MS treatment of both, the present trial was designed as a four-group randomized controlled trial to observe MS of the S3 nerve root, M1 area, M1 area + S3 nerve root. In order to better compare the feasible effects of each trial protocol, the feasible effects of combined MS of M1 and S3 nerve roots can be compared, and a sham stimulation group was designed to validate the feasible effects of MS in the treatment of patients with urinary retention after SCI.

In this trial, the bladder residual urine volume will be used as the primary assessment index, and bladder residual urine volume can only quickly, intuitively and objectively assess the patient’s residual urine volume situation with lower cost. Urinary diary as a secondary index can reflect the patient’s urination situation for 3 consecutive days before and after treatment, which is a more comprehensive assessment of the patient’s overall improvement in urination. fNIRS is a functional brain imaging technique based on hemodynamics, and its principle is similar to fMRI, which is widely used to study the central control of bladder function, anorectal function and sexual function, but it is expensive and inflexible, while fNIRS has the advantages of good portability, high temporal resolution and low cost. The advantages of using fNIRS to explore the mechanism of action of central control of bladder function is obvious in this trial, and no study have been found using fNIRS examination to explore the central control of bladder function after SCI. Therefore, it is important to study the recovery of bladder function in patients with urinary retention after SCI based on fNIRS technology. Pelvic floor sEMG Glazer assessment can reflect pelvic floor muscle function, and pelvic floor muscle dysfunction can seriously affect bladder function, so it is important to assess pelvic floor muscle function; QoL score is a subjective perception of the impact of bladder symptoms on the QoL of patients, which reflects the degree of patient satisfaction with the treatment effect to a certain extent. This trial used urinary ultrasound, fNIRS, electrophysiology, scale scores, and other assessment indicators, to comparative objective and comprehensive evaluation of the feasibleeffects of different protocols for the treatment of urinary retention after SCI, which has important clinical significance for the rehabilitation of patients with urinary retention after SCI.

In this trial, four groups of RCTs were used to compare the therapeutic feasible effects of peripheral, central MS and peripheral combined central MS, so as to propose a more desirable therapeutic plan and explore the possible mechanism of action by combining fNIRS and electrophysiology assessment methods, which can provide more reliable reference for the rehabilitative treatment of urinary retention after SCI.

## Limitations

Despite the above advantages of this study, there are still some limitations. First, the patients we included were only with urinary retention after SCI, so our conclusions are only applicable to patients with urinary retention after SCI. Second, due to the lack of professional evaluation of patients’ psychological state, the influence of psychological state on the therapeutic effect is neglected. In addition, the inclusion of subjects in this study was strictly controlled, but the heterogeneity of patients in the course of experimental intervention should still be taken into account, such as the different dose of drug intervention and the different speed of nerve recovery in patients of different ages. Nevertheless, heterogeneity was similar across conditions due to the use of randomized controlled designs.

## Author contributions

LD: Writing – original draft, Supervision. XT: Writing – review & editing. CG: Writing – review & editing. YiL: Writing – review & editing. LX: Writing – review & editing. YuL: Writing – review & editing. MW: Writing – review & editing. YZ: Writing – original draft.

## Supplementary material

SUPPLEMENTARY MATERIAL Informed consent form (Chinese).
