# Supplementary material for: Effects of central-peripheral FMS on urinary retention after spinal cord injury: a pilot randomized controlled trial protocol
Source: Front Neurol. 2024 Jan 5;14:1274203. doi: 10.3389/fneur.2023.1274203 (PMC10797019; doi:10.3389/fneur.2023.1274203)
Supplement: SUPPLEMENTARY MATERIAL — Informed consent form (Chinese). [file Data_Sheet_1.doc]

**知情同意书**

（受试者须知页）

方案名称：“中枢-外周”重复磁刺激对脊髓损伤后尿潴留的临床疗效研究

研究者：钟燕彪、王茂源、董凌燕、陶茜、龚成

申办者:赣南医学院第一附属医院

尊敬的受试者：

您被邀请参与“中枢-外周”重复磁刺激对脊髓损伤后尿潴留的临床疗效研究，该项目由赣南医学院第一附属医院提供支持。请仔细阅读本知情同意书并慎重做出是否参与本研究项目的决定。作为受试者，您需在加入科学研究前签署您的纸质版知情同意书。当您的研究人员和您讨论知情同意书的时候，您可以让他/她给您解释您看不明白的地方。我们鼓励您在做出参与此研究项目的决定之前，和您的家人及朋友进行充分讨论。您有权拒绝参加本研究，也可随时退出研究，您应有的权利不会因此受到损害。若您正在参与其它研究项目，请告知您的研究人员。本研究的背景、目的、研究过程及其他重要信息如下：

# 一、研究背景

脊髓损伤(SCI)是由外界因素造成的脊髓功能和结构损坏，从而出现各种运动、感觉、自主神经功能障碍，而随后出现的并发症，以尿频、尿急、尿失禁、尿潴留、遗尿等下尿路症状为主要表现，尿潴留是SCI后神经源性膀胱最常见的并发症，需要长期留置导尿管，给患者生活带来极大不便。因此，改善膀胱功能、促进自主排尿对减轻尿潴留程度和提高患者生活质量是脊髓损伤康复的重要组成部分。

近年来，随着磁刺激越来越多的应用于临床，其疗效及安全性得到了国内外众多学者验证，给NB患者提供了一种新的治疗途径。磁刺激治疗主要以调节支配膀胱尿道的中枢神经及周边神经的抑制性和兴奋性来调节膀胱功能。FMS是一种无损伤、简单易行的方法，能显著改善排尿功能障碍患者的症状，提高患者的生活质量。目前临床上 rMS治疗脊髓损伤后神经源性膀胱常用的部位有骶神经根、盆底会阴部、耻骨上膀胱区等 , 其中以刺激骶神经根较多见。当线圈作用于骶神经根时,刺激 S2-S4神经根孔,以S3神经根孔多见。最新研究表明,脊髓、骨盆区域相对应的皮层区域也可作为刺激部位。大量得研究表明，不同部位的磁刺激治疗脊髓尿潴留均有效，但是不同部位磁刺激哪个疗效更优尚不明确，因此，本研究旨在观察通过功能性磁刺激不同部位治疗脊髓损伤后神经源性膀胱尿潴留的临床效果。

# 二、 研究目的

本研究旨在观察通过功能性磁刺激刺激中枢和外周治疗脊髓损伤后神经源性膀胱尿潴留的临床效果

# 三、研究过程

1. **多少人将参与这项研究？**

大约60人将在相同的医疗机构内参与本项研究。

# 研究步骤

如果您同意参加本研究，请您签署这份知情同意书。在您入选研究前，医生将询问、记录您的病史，必要时进行心电图、血常规、尿常规、尿动力学检查、泌尿系彩超等筛选检查。根据纳入及排除标准并对您进行初步的筛选，确定您可以参加本研究后，将对您的病情进行必要的评估，研究将根据随机数字表进行随机分配到4个治疗组： A组:假刺激组；B组：经颅磁刺激疗法；C组：磁刺激骶神经根，D组：在C组的基础上再给予经颅磁刺激治疗。整个治疗周为期 2周，每周5次，每日1次，常规康复治疗根据患者实际情况由康复治疗师进行，分别在治疗前、后进行脑功能影像学评估（fNIRS）、测量膀胱残余尿量、排尿情况变化及行Glazer评估盆底肌表面肌电值变化情况、生活质量评分的评估，最后汇总数据进行统计分析。

# 这项研究会持续多久？

2周。您可以在任何时间选择退出研究。如果在研究途中您决定退出，我们建议您先和您的研究人员商议。在研究过程中，如果您出现严重的不良事件，或者您的研究人员觉得继续参加研究不符合您的最佳利益，他/她会决定让您退出研究。申办者或者监管机构也可能在研究期间终止研究。但您的退出不会影响您的正常诊疗权益。如果您因为任何原因从研究中退出，您可能被询问有关您参加研究的情况。如果研究人员认为需要，您也可能被要求进行有关实验室检查和体格检查。

# 研究中收集的信息和生物标本

在治疗前及结束后均为您进行脑功能影像学评估（fNIRS）、测量膀胱残余尿量、排尿情况变化及行Glazer评估盆底肌表面肌电值变化情况、生活质量的评估，所有信息将以CRF表的形式保存在研究项目负责人手中。

# 四、风险与受益

1. **参加本研究的风险是什么？**

参加本研究可能给您带来的风险如下。您应该和您的研究人员或者与平日照看您的医生讨论一下这些风险。研究期间，您可能会发生一些、所有或者不发生这些不良事件、风险、不适、不方便，如头晕、耳鸣等。如您出现任何不适，或病情发生新的变化，或任何其他意外情况，不管是否与研究有关，均应及时通知您的研究人员，他/她将对此作出判断并给与适当的医疗处理。您在研究期间需要按时到医院随访，做一些相关检查，这将会占用您的一些时间，也可能给您造成麻烦或带来不方便。

# 参加研究有什么受益？

您将在本项研究中可能受益，您将在常规监测以外得到细致的评估、监护与治疗,您的病情有可能获得改善。

# 五、备选的治疗方案

除了参与本研究，您可以接受您的医生提供的常规康复治疗，如针灸、作业治疗等。请您和您的医生讨论这些及其他可能的选择。

# 六、研究结果的使用和个人信息的保密

在您和其他受试者的理解和协助下，本项目的研究结果可能会在医学杂志上发表，我们会按照法律的要求为您的研究记录保密。研究受试者的个人信息将受到严格保密，除非应相关法律要求，您个人信息不会被泄露。必要时，政府管理部门和医院伦理委员会及其它相关研究人员可以按规定查阅您的资料。

# 七、关于研究费用及相关补偿

1. **研究所用的药物/器械及相关检查费用**

研究过程中会免除经颅磁刺激治疗及所有相关评估项目的费用，根据病情必要时行尿动力学检查相关评估的费用将有您承担。其他常规康复治疗手段是目前临床诊疗过程中常实施的项目，因此，这些项目的费用将由您支付(如是医保支付范围可由医保支付)。对于您同时合并的其他疾病所需的治疗和检查，也将由您自行支付。

# 参加研究的补偿

为参与本研究所花费的开支（如您的交通费和额外采血的营养费），没有额外的补偿。

# 发生损伤后的补偿/赔偿

经颅磁刺激治疗为非侵入性脑刺激技术，目前临床以广泛应用，一般无损伤及副作用，如果发生与该项研究相关的损伤，您将获得相关治疗，并按中国有关法律进行补偿/赔偿。

# 八、受试者的权利和相关注意事项

1. **您的权利**

在参加研究的整个过程中，您都是自愿的。如果您决定不参加本研究，不会影响您正常的诊疗权益。如果您决定参加，请您在这份书面知情同意书上签字。您有权在试验的任何阶段随时退出试验而不会遭到歧视或受到不公平的待遇， 您相应医疗待遇与权益不受影响。

# 注意事项

作为受试者，您需要提供有关自身病史和当前身体状况的真实情况；告诉研究医生自己在本次研究期间所发现的任何不适；不得服用医生已告知的受限制药物、食物等；告诉研究医生自己目前或最近是否参与其他研究。

# 九、获知信息的相关联系方式

如果在研究过程中有任何重要的新信息，可能影响您继续参加研究的意愿时， 您的研究人员将会及时通知您。如果您对自己的研究数据，或研究结束后您希望知道本研究的发现,您可联系董凌燕医生:18270712563,医院科研伦理委员会已审查通过该研究项目，如果您有与自身权利/权益相关的任何问题，或者您想反映参与本研究过程中遭遇的困难、不满和忧虑，或者想提供与本研究有关的意见和建议，请联系赣南医学院第一附属医院科研伦理委员会，联系电话：0797-8689034，电子邮件：gyfykjk@163.com.
